# Archaeal phylogenomics provides evidence in support of a methanogenic origin of the Archaea and a thaumarchaeal origin for the eukaryotes

**DOI:** 10.1098/rspb.2010.1427

**Published:** 2010-09-29

**Authors:** S. Kelly, B. Wickstead, K. Gull

**Affiliations:** 1 Sir William Dunn School of Pathology, University of Oxford, South Parks Road, Oxford OX1 3RE, UK; 2 Centre for Mathematical Biology, University of Oxford, 24–29 Saint Giles', Oxford OX1 3LB, UK; 3 Oxford Centre for Interactive Systems Biology, University of Oxford, South Parks Road, Oxford OX1 3QU, UK; 4 Department of Plant Sciences, University of Oxford, South Parks Road, Oxford OX1 3RB, UK

**Keywords:** Archaea, Eukarya, methanogenesis, evolution, Bacteria

## Abstract

We have developed a machine-learning approach to identify 3537 discrete orthologue protein sequence groups distributed across all available archaeal genomes. We show that treating these orthologue groups as binary detection/non-detection data is sufficient to capture the majority of archaeal phylogeny. We subsequently use the sequence data from these groups to infer a method and substitution-model-independent phylogeny. By holding this phylogeny constrained and interrogating the intersection of this large dataset with both the Eukarya and the Bacteria using Bayesian and maximum-likelihood approaches, we propose and provide evidence for a methanogenic origin of the Archaea. By the same criteria, we also provide evidence in support of an origin for Eukarya either within or as sisters to the Thaumarchaea.

## 1. Introduction

All cellular life on this planet belongs to one of three distinct domains: the Eukarya, the Bacteria and the Archaea [1]. Since their inception as domains, their inter-relationship and evolutionary history has been a focus of debate (for reviews see [2–5]). However, some crucial details impose directionality on the course of evolution. The most important fact is that the common ancestor of all modern-day eukaryotes contained an endosymbiont, which originated from an α-proteobacterium [4,6–9]. This α-proteobacterial endosymbiont evolved to become the mitochondrion, mitosome and hydrogenosome of all extant eukaryotes [10,11], a fact that clearly establishes a temporal order which requires that the major lineages of the Bacteria arose before the appearance of the last common ancestor of all extant eukaryotes. While the identity of the host cell which adopted this endosymbiont and the relationship between Bacteria, Archaea and Eukarya remains ardently disputed [4,6,12–14], the relationship of organisms within the three major domains is gradually becoming clearer.

Elucidating genome content and determining gene ancestry have been decisive factors in inferring the major events in the evolution of life on the Earth. This type of analysis is particularly important for unicellular organisms where discernable morphological markers are inherently less numerous. Determining a pattern of relationship between all organisms based on these data has become a goal of post-genome era evolutionary analyses. However, attempts at defining such relationships are confounded by both methodological problems—such as inadequate models of sequence evolution and methods of tree inference—and lack of information both in terms of numbers and evolutionary distribution of sequenced genomes. Even with the expanding availability of genome information, it has become clear that the most significant barrier facing the construction of a hierarchical tree, if one can truly be considered to exist, is lateral gene transfer [3,15,16]. Discerning the true evolutionary history of life will require untangling what has been vertically inherited from what has been laterally acquired.

Among the big questions facing evolutionary biologists today are the origin of cellular life and the subsequent emergence of modern-day eukaryotes. Regarding the latter, an initial and popular view was that genes in the eukaryotic lineage with detectable bacterial or archaeal homology can be split into two groups. Genes associated with operational functions such as metabolism and biosynthesis were thought to be predominantly bacterial in origin, whereas those associated with informational processes, such as transcription, translation and replication originated from the Archaea [7,12,17,18]. However, with the exception of the cyanobacterial genes found in plants [19,20] and the α-proteobacterial genes acquired from the mitochondrion [7,8,20], this initial view has more recently been called into question. Recent interrogations of the source of many eukaryotic operational genes suggest surprisingly different origins from within both Bacteria and Archaea [13]. Though the origin of the informational genes is less ambiguous, displaying an almost exclusively archaeal ancestry, the identification of the precise archaeal lineage involved remains elusive.

Previous studies have shown that several eukaryotic informational genes, such as DNA pol D [6], eukaryote-like histones [21,22] and cell division protein FtsZ [23], are found only in one particular branch of the Archaea known as the Euryarchaea. These observations suggested a root for eukaryotes either within or as sisters to the Euryarchaea—a hypothesis that was supported by SuperTree analyses which placed Eukarya within the Euryarchaea as sisters to Thermoplasmatales [20]. Other large multi-gene approaches have rendered hypotheses that the eukaryotes descend from an ancient and uncharacterized archaeal lineage [12]. However, most phylogenetic reconstructions using informational gene sequence data recover a root for the eukaryotes in the Crenarchaea: known as the ‘eocytes’ hypothesis [13,14,24–30]. This multitude of conflicting analyses has, in part, inspired the proposal of a number of different hypotheses. Popular among these are hypotheses which state that the eukaryotes are not derived, in particular, from any group of Archaea, but are instead a sister group to the Archaea as a whole, sharing a common or ‘neomuran’ ancestor [5,6,31]. There are also other hypotheses which submit that primitive endosymbiont-lacking pre-eukaryotes were the first cellular organisms [32,33], evolving before both Bacteria and Archaea.

To complicate issues further, all of the above phylogenetic analyses of selected protein genes, ribosomal RNA (rRNA) genes and gene synapomorphies evoke origins for cellular life which appear incompatible with theories on the evolution of biochemistry [34–36]. Popular among these is the idea that acetogenesis and methanogenesis were the ancestral forms of energy metabolism in the earliest free-living Bacteria and Archaea, respectively [34,35]. These theories also propose that these biochemistries evolved under conditions similar to those found today in deep sea alkaline hydrothermal vents, forming the energy currency which funded the emergence of the RNA world [37].

Here, we use the new wealth of genome information to address the relationship between the three domains of life using iterative hidden Markov model (HMM) gene-family finding algorithm to identify 3537 discrete orthologous protein families within the Archaea. We then provide a novel approach for the interrogation of the inter-relationship of this dataset with both the Eukarya and Bacteria using both Bayesian and maximum-likelihood approaches.

## 2. Material and methods

### (a) Identification of discrete orthologous groups

The sources and versions for genome projects used in this study are given in electronic supplementary material, S1. Iterative searches were performed for each of the 104 759 predicted protein sequences contained in the 48 selected fully sequenced archaeal genomes. Each sequence was subject to the same search criteria. To initiate each iterative search, a single sequence was converted to a HMM and used to search all 48 genomes using the Hmmer program [38]. The resultant hits were filtered based on an *e*-value threshold of 1 × 10^−5^ and aligned using MAFFT [39]. Columns within the alignment that contained more than 50 per cent gaps were removed to prevent species-specific or clade-specific amino acid insertions biasing the models. These gap-parsed alignments were then further parsed to remove sequences with greater than 95 per cent identity to any other sequence within the alignment. This step was carried out to prevent biasing of the HMM towards any particular group of organisms, which may be over-represented in the alignment owing to the presence of paralogues. This parsed alignment was then used to generate the HMM for the next round of searches. Searches were terminated when no further hits passing the *e*-value threshold could be identified. The results from the individual searches were then analysed. To be considered a discrete orthologous group (DOG), we then stipulated that the search results for each member of a group had to recover the entire group exclusively. If the search results did not agree then the group was discarded. This resulted in the identification of 3537 DOGs. To analyse the distribution of these gene groups outside Archaea, the HMMs for each of the groups were used to independently search a set of genome sequence from 29 eukaryotes and 29 bacteria. The eukaryotic and bacterial genomes selected are also listed in electronic supplementary material, S1. Where multiple paralogous genes were found in any eukaryote or bacterium, the highest scoring gene was selected to be included in the final alignment.

### (b) Phylogenetic inference

To infer binary data trees, the detection/non-detection data from the 3537 DOGs were converted to binary data and analysed using the program MrBayes v. 3.1.2 [40]. One thousand re-sampled replicates of each tree inference were run using the restriction (binary) model to increase the robustness of the analysis. In each case, a *γ*-distributed rate variation was approximated by eight discrete categories with shape parameter estimated from the data. The ‘covarion’ model [41] was also implemented to allow characters invariant in one clade to be variable elsewhere in the tree. Four chains were employed, each with a temperature of 0.2. Each inference was made from a random start tree and allowed to run for 300 000 generations. The time taken to reach stationary phase was between 7000 and 15 000 generations per replicate. The final 200 000 trees sampled every 500 generations from each of the 1000 replicates were used to construct the consensus bootstrapped Bayesian tree.

Amino acid sequence alignments were produced for each of the 3537 DOGs using MAFFT, these were trimmed using Gblocks [42] to remove poorly aligned positions, which may not be homologous or may have been saturated by multiple substitutions. These parsed alignments were concatenated together to produce a single alignment of 694 908 aligned positions. In order for this alignment to fit into 12 Gb of computer memory, the alignment was further trimmed to contain only positions containing 10 or more non-gap characters, producing a final alignment of 44 703 positions. This final alignment was used to infer Bayesian trees using the program MrBayes v. 3.1.2. Four re-sampled replicates of each tree inference were run using each of the WAG, Dayhoff and Blossum substitution matrices. In each case, a *γ*-distributed substitution rate variation was approximated by four discrete categories with shape parameter estimated from the data. The ‘covarion’ model was implemented as above. Four chains were employed, each with a temperature of 0.2. Each inference was made from a random start tree and allowed to run for 300 000 generations. The time taken to reach stationary phase was approximately 20 000–50 000 generations per replicate (stationary phase was manually determined by examining traces in the .p files). The final 200 000 trees sampled every 500 generations from each replicate were used to construct the consensus multi-model Bayesian tree. The same concatenated alignment was also used to infer a 100 bootstrap replicate maximum likelihood tree using the using the RAxML v. 7.0.4 program [43] using the blosum62 amino acid matrix with site-specific evolutionary rates approximated by four discrete *γ* categories.

### (c) Interrogation of the intersection of the archaeal tree

To interrogate the position of the intersection of the archaeal tree with both the eukaryotic and bacterial trees, we modified a version of MrBayes v. 3.1.1 to allow us to constrain branching order while allowing branch lengths to vary. Since topology is constrained, this approach allows us to place the intersection at any position in the archaeal tree and evaluate the overall likelihood of that tree once the other parameters have been estimated. In all cases, the branching order of the archaeal tree was constrained to the order recovered from the large concatenated amino acid alignment multi-model Bayesian tree described above. Two experiments were performed: the first evaluated the likelihood of each of a set of trees, where each tree was an unrooted tree which had the eukaryotes as a monophyletic group intersecting with a specific branch of the archaeal tree. The second experiment was similar to the above but with the bacteria included as the monophyletic group. The branching order of the 29 eukaryotic organisms used in this analysis was constrained according to the consensus of recent analyses derived from rRNA, organellar-genome and concatenated multi-gene phylogenetic analyses [44–46] with the root positioned between the unikonts and bikonts. Similarly, the branching order of the 29 selected bacteria was constrained according to the consensus of previous whole genome and large concatenated sequence analysis of carefully selected orthologues [47,48]. For the purpose of this analysis, both Bacteria and Eukaryota are assumed to be independent monophyletic groups. Forty-seven tree topologies were created, one for each non-terminal branching event in the archaeal tree and one for each of the two longest branches (those leading to candidatus *Korarchaeum cryptophylum* and *Nanoarchaeum equitans*, respectively). Each tree evaluation was run using each of the WAG, Dayhoff and Blossum substitution matrices. In each case, a *γ*-distributed substitution rate variation was approximated by four discrete categories with shape parameter estimated from the data. Each inference was allowed to run for 50 000 generations. The time taken to reach stationary phase was approximately 10 000–20 000 generations (stationary phase was manually determined by examining traces in the .p files). The tree hypothesis with the best marginal likelihood was selected and log 10 Bayes factors were calculated for all sub-optimal trees. For calculating log 10 Bayes factors, we sampled the final 20 000 trees every 500 generations from each inference. The log 10 Bayes factors [49–51] were calculated using the Tracer program [52] with modifications proposed by Suchard [53]. The average of the log 10 Bayes factors for each substitution model was used to specify the colour of the heat map in figure 2. Unconstrained trees for each intersection dataset were also inferred. These trees were each composed from a 100 bootstrap replicate maximum likelihood tree using the RAxML v. 7.0.4 program [43] using the blosum62 and WAG amino acid matrices with site-specific evolutionary rates approximated by four discrete *γ* categories.

### (d) Shimodaira–Hasegawa test

To provide support for the Bayes factor analyses via an independent method, we performed an analogous test using a maximum-likelihood approach: the Shimodaira–Hasegawa (SH) test [54]. Using the same alignments as used for the Bayes factor analysis, we compared the most likely tree from the Bayes factor analysis to all other trees interrogated in the intersection tests. The SH tests were implemented using RAxML v. 7.0.4 [43] implementing the PROTGAMMAWAG model of amino acid substitution. For ease of display, all likelihood difference values were normalized to the most likely value. To support these findings, the approximately unbiased (AU) test of regions using multi-scale bootstrap resampling was also performed [55].

## 3. Results

### (a) Identification of 3537 discrete orthologue groups

To look at the evolution and inter-relationship of the three domains of life, we started by identifying a set of DOGs in the Archaea. We define a DOG as a group of related sequences which contains no more than one sequence from any one taxa. Iterative profile-based searches were performed for each of the 104 759 predicted protein sequences contained in the 48 selected fully sequenced archaeal genomes. This search procedure produces three categories of result: (i) no sequences are identified apart from the initial query sequence (*n* = 18 197); (ii) more than one sequence identified but no more than one sequence per genome (*n* = 20 181); and (iii) multiple sequences in at least one genome (*n* = 66 381).

Searches which failed to return sequences in addition to the query sequence (category 1) contain no phylogenetic information and were hence discarded. Searches that produced paralogous gene families in one or more Archaea (category 3) were also discarded, as it is often difficult to extract useful phylogenetic information from paralogous families. Searches that identified only single orthologues in any given archaeal genome (category 2) were retained for further analysis. The results from each of the retained searches were compared and only groups which were recovered consistently by queries initiated with any member sequence were then kept for further analysis. This final set, in which no sequence appears more than once, comprises 3537 DOGs (electronic supplementary material, S2 and S3). Though there is a possibility of hidden paralogy within this dataset, all DOGs are, from the point of view of this analysis, considered to represent true orthologous gene families. The average number of DOGs obtained per archaeal genome was 430, s.d. = 137 (electronic supplementary material, S3).

**Figure 1.**
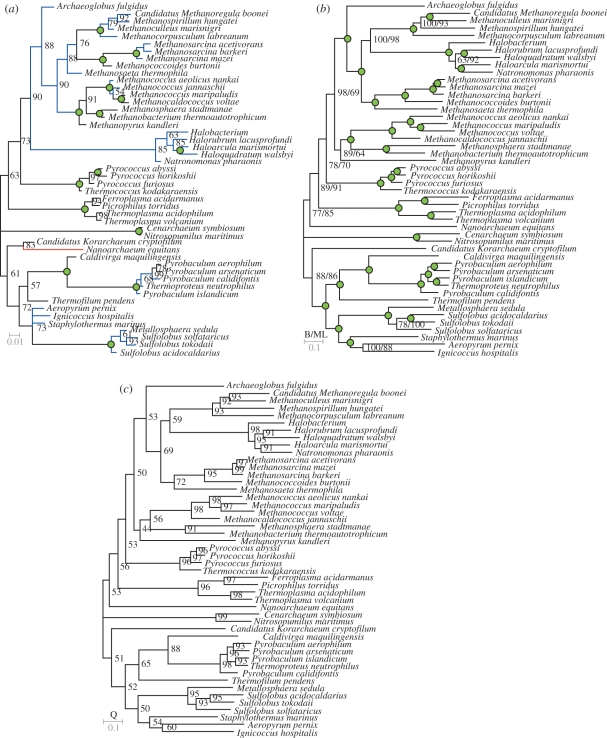
Unrooted phylogenetic trees inferred using DOG data. (*a*) Bootstrapped Bayesian phylogenetic tree inferred using detection and non-detection data. Black branches indicate branches that agree with the protein sequence tree, blue branches indicate that the grouping is correct but the order differs from the protein sequence tree and red branches indicate that the branch is in a different position in the protein sequence tree. Values at nodes represent bootstrap values. Green circles indicate 100% bootstrap support. (*b*) Multi-model Bayesian phylogenetic tree inferred using concatenated protein sequence. Green circles indicate 100% support under all methods. Values at nodes provided when support is less than 100%. Bayesian and maximum-likelihood values indicated by B and ML, respectively. (*c*) Quartet analysis support for the concatenated protein sequence phylogeny. Values at nodes represent percentage support from quartet analysis. In all cases, scale bars indicate number of changes per site.

Using the detection/non-detection data as binary phylogenetic characters, we inferred a bootstrapped Bayesian phylogeny (figure 1*a*). This produces a phylogeny which closely matches current opinion on archaeal phylogeny based on concatenated protein sequence alignments [13,56]. The binary-data tree also consolidates the hypothesis, based on aligned protein sequence data, that the Thaumarchaea (including *Nitrosopumilis maritimus* in our analysis) forms an independent group distinct from the Crenarchaea or Euryarchaea [56]. There are only two notable differences between this phylogeny and current opinion on archaeal phylogeny. The first is the position of *Nanoarchaeum equitans*, a symbiotic/parasitic archaeaon which has undergone large-scale genome reduction during its evolution [57,58]. In our analysis, *N. equitans* contains only 105 DOGs which is less than a quarter of the average number. The other difference is the position of the Halobacteria. Although the Halobacteria themselves are not methanogenic, their consistent position within previously reported phylogenies indicates that their ancestor was a methanogen, which subsequently lost the ability to produce methane. The position of the Halobacteria within the binary-data tree as an outgroup to the methanogens is hence reflective of its biology and the concomitant loss of genes involved in methanogenesis. The congruence of this binary tree with current thinking on archaeal phylogeny demonstrates that the distribution of the majority of these genes is consistent with a pattern that specifies non-lateral inheritance. This detection/non-detection data were also analysed using bootstrapped split-decomposition and bootstrapped neighbour-net phylogenetic network methods [59]. Under both the methods, the major phylogenetic groupings of the Archaea are recovered with high confidence intervals (electronic supplementary material, S4). This congruence between networks and phylogeny provides further support for the direct non-lateral inheritance of the majority of the DOGs in our dataset and indicates that, for this set, there has been no significant lateral transfer of genes.

### (b) Concatenated DOG protein sequence alignments yield well-supported phylogeny

We sought to use the large quantity of information contained in the DOGs to perform a robust phylogenetic analysis of the Archaea. We used the concatenated protein sequence alignments from the 3537 DOGs to infer Bayesian phylogenetic trees using the WAG, Dayhoff and Blossum amino acid substitution matrices. This large multi-protein dataset contained 44 703 aligned positions in 10 or more taxa. The tree obtained from this analysis (figure 1*b*), which contains both more taxa and more aligned positions than previous analyses, is in agreement with the current thinking on archaeal phylogeny [13,56]. We also provide support for these Bayesian trees by a bootstrap maximum likelihood tree (figure 1*b*). To provide further support for these concatenated sequence phylogenies, the individual amino acid sequence alignments for each DOG were also each subject to tree inference without concatenation. The resulting consensus trees from each inference were split into their constituent (all possible) quartets and each quartet was compared with the concatenated-sequence phylogeny. Only one node in the concatenated-sequence phylogeny received less than 50 per cent support by this method (figure 1*c*). Hence this archaeal phylogeny is robust, being both independent of the method of tree inference and the model of amino acid substitution employed. As our phylogeny is supported by quartet analysis, it demonstrates that it is not subjected to effects caused by missing data within our concatenated alignments.

### (c) Identification of conserved DOGs in bacteria and eukaryotes

We used the HMMs for each of the 3537 DOGs to identify homologues in the genomes of 29 eukaryotes and 29 bacteria. We were able to detect homologues of 320 and 463 DOGs in eukaryotes and bacteria, respectively. We used the concatenated protein sequence alignments from the 320 (electronic supplementary material, S5) and 463 (electronic supplementary material, S6) DOGs conserved in Eukarya and Bacteria, respectively, to interrogate the position of the intersection of these two domains with the Archaeal tree. These alignments were parsed in the same manner as described above to produce datasets of 25 069 and 33 516 aligned positions, respectively.

Two independent sets of tests were performed to interrogate the position of the intersection in the Archaeal tree with either Bacteria or Eukarya. In both tests, the marginal likelihood was evaluated for each of a set of trees, where each tree was a topologically constrained tree, which had either the eukaryotes or the bacteria as a monophyletic group intersecting with a particular branch of the archaeal tree constrained from our analysis above. The tree hypothesis with the best marginal likelihood was selected and log 10 Bayes factors were calculated for all sub-optimal trees using three different models of substitution (Dayhoff, Blossum and WAG). The log 10 Bayes factors recovered under each substitution model exhibit very highly significant linear relationships (*r*^2^ > 0.99, *p* < 0.00001 in all cases) and are hence independent of the amino acid substitution model employed (electronic supplementary material, S7). Therefore, this method of analysis overcomes any problems arising from systematic error introduced by assuming a particular amino acid substitution model and is presented here as an alternative to a model-fitting approach.

**Figure 2.**
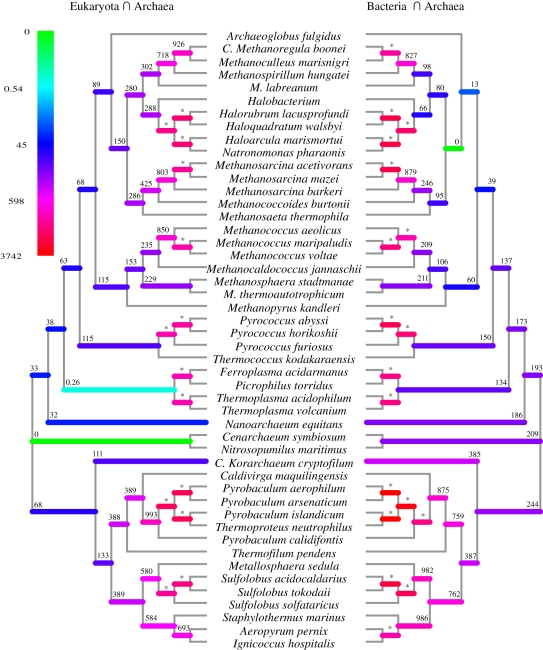
Log 10 Bayes factor analysis of intersection position in the archaeal tree determined using eukaryotic and bacterial data. Cladogram of unrooted archaeal tree as shown in figure 1. Colour of branches indicates average log 10 Bayes factor for this intersection position. Heat map for log 10 Bayes factors is provided, the colour scheme goes from green (most likely) through blue to red (least likely). Log 10 Bayes factors are given above branches. Asterisks (*) indicate a log 10 Bayes factor of over 1000.

Testing the intersection of the Archaea with the Bacteria revealed that the most likely intersection occurs on the branch which separates the mesophilic methanogens and Halobacteria from the hyperthermophilic methanogens (figure 2). This result is dramatically different from the previous analyses of Archaea and Bacteria based on rRNA sequences [1]. As it has been previously reported to various extents that there has been lateral gene transfer between the mesophilic Archaea and Bacteria [60–62], we performed an additional test on the archaeal–bacterial intersection. This analysis was as above but with all DOGs found only in the Halobacteria, mesophilic or methanogenic Archaea and Bacteria removed (removed *n* = 109). The removal of these DOGs produced no effect on the distribution of the log 10 Bayes factors (electronic supplementary material, S8 and S9). Hence, the location of the signal is not attributable to genes shared only between methanogens, halophiles or mesophiles and Bacteria. While many of the genes which are potentially laterally transferred are removed by this step, it is possible that there is still some laterally transferred information remaining.

The point in the archaeal tree which intersects with the eukaryotes is predicted to be most likely in the region where the Thaumarchaea branched from the euryarchaeal line. The most likely intersection point is on the branch leading to *Cenarchaeum symbiosum* and *Nitrosopumilis maritimus* (figure 2), as seen in one previous analyses [56]. The second most likely intersection point was on the branch leading to the Thermoplasmatales. This second intersection was previously identified as the strongest archaeal signal in eukaryotic genomes in a large SuperTree interrogation [20] and is a result which is consistent with previous large-scale analyses [12]. In addition to this, we found that the three next most likely intersections are all euryarchaeal, suggesting that the true intersection, if not in the Thaumarchaea, is likely to be in the region where the Thaumarchaea and Euryarchaea diverged. Unconstrained trees for each intersection dataset were also inferred using a bootstrapped maximum-likelihood method. These trees support the positions of our most likely intersections (electronic supplementary material, S10). Additional support is also provided by the analysis of an enriched dataset of eukaryotic DOGs, which may have bacterial origins (electronic supplementary material, S11).

### (d) Maximum-likelihood support for the Bayesian tests

**Figure 3.**
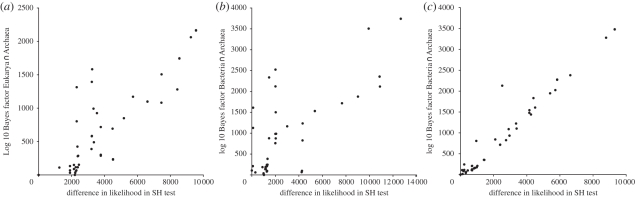
Comparison of difference in log-likelihoods obtained via SH test with log 10 Bayes factors inferred from the same data. In both cases, the tree hypothesis with the best log-likelihood value was selected and log 10 Bayes factors or difference in log-likelihood was calculated for all sub-optimal trees. (*a*) Comparison using Eukarya ∩ Archaea data (*r*^2^ = 0.709, *p* < 0.0001). (*b*) Comparison using Bacteria ∩ Archaea data (*r*^2^ = 0.614, *p* < 0.0001). (*c*) Comparison after removing DOGs shared between Halobacteria, methanogens and Bacteria (*r*^2^ = 0.950, *p* < 0.0001).

To provide support for the Bayesian tests above using a methodologically distinct approach, we performed SH tests [54] using maximum likelihood. In the case of the eukaryotes, this independent test agrees that the most likely intersection point lies on the branch leading to the Thaumarchaea. This hypothesis is significantly better than all other tree hypotheses under both the SH and AU test (*p* < 0.0001; electronic supplementary material, S14). There is also a significant correlation (*r*^2^ = 0.709) between the log 10 Bayes factor obtained for a given node and the difference in log-likelihood between that node and the most likely node under the SH test (figure 3*a*). In the case of the Bacteria, there is a weaker correlation (*r*^2^ = 0.614) between the log 10 Bayes factor obtained for a given node and the difference in log-likelihood between that node and the most likely node under the SH test (figure 3*b*). Moreover, the most likely node recovered under the Bayesian analysis is only the ninth most likely in the SH test analysis. The first six are not significantly better than each other by SH test but are significantly different by the AU test (*p* < =0.001; electronic supplementary material, S14). Interestingly, each of the nodes which produce better log-likelihood values in the SH analyses (when compared with the Bayesian analysis) are confined to one particular branch of the archaeal tree. This branch contains the *Methanopyrus kandleri*, *Methanobacterium thermoautotrophicum*, *Methanospaera stadtmanae*, *Methanocaldococcus jannaschii, Methanococcus voltae, Methanococcus maripaludis* and *Methanococcus aeolicus nankai*. However, when we repeat the SH test on the dataset in which DOGs found only in the methanogenic Archaea and Bacteria have been removed, we find that this discrepancy between test methods disappears (figure 3*c* and electronic supplementary material, S14). Additionally, there is now a strong linear correlation between the log 10 Bayes factor obtained for a given node (under both the full and reduced dataset analyses) and the difference in log-likelihood between that node and the most likely node under the SH test (*r*^2^ = 0.950 and *r*^2^ = 0.945, respectively). Moreover, this analysis agrees with the Bayesian analysis in that placement of the most likely intersection point in the archaeal tree is at the base of the mesophilic methanogens. The *p*-values for the SH and AU tests and the corresponding log 10 Bayes factors for all of the above analyses are shown in electronic supplementary material, S14.

### (e) Compositional heterogeneity

To analyse whether there was any correlation between compositional heterogeneity and the log 10 Bayes factors produced in the above intersection tests, we performed several independent tests. In the case of the intersection of the archaeal dataset with the eukaryotes (electronic supplementary material, S12 part A), there is no significant correlation (*r*^2^ = 0.0297, *p* = 0.8766) between a more ‘Eukaryote-like’ amino acid composition and the log 10 Bayes factor obtained for a given node in the archaeal tree. This analysis also shows that the most likely intersection point in the Archaea is not that most compositionally similar node to the eukaryotes. Similarly, for intersection of the archaeal dataset with the Bacteria (electronic supplementary material, S12 part B), the most likely intersection is not the most compositionally similar node to the Bacteria. However, unlike the eukaryotes, there is a weak correlation (*r*^2^ = 0.2025, *p* = 0.0008) between the log 10 Bayes factor obtained for a given node and similarity between composition profile of that node and the composition profile of the Bacteria.

### (f) Addressing the effect of the proportion of gap-characters

In studies of this type, it is important to consider the effect of the proportion of gap-characters present in the multiple sequence alignments, frequently referred to as ‘missing information.’ In the large concatenated alignment which was used to infer the phylogeny of the Archaea in figure 1*b*, the mean proportion of ‘missing data’ was 44 per cent. However, we demonstrated that this tree is not subject to effect caused by missing data by providing quartet analysis support. To interrogate whether reduction in the amount of ‘missing data’ has an effect on the observed likely intersection points, we re-executed each of the above intersection tests using a more stringent cut-off for missing data inclusion (electronic supplementary material, S13), reducing the proportion of ‘missing data’ from 55 and 64 per cent to 12 and 11 per cent in the case of the eukaryotes and Bacteria, respectively. In the case of the eukaryotes, we show that there is little effect on the distribution of the log 10 Bayes factors (*r*^2^ = 0.975, *p* < 0.0001) and no effect on the position of the most likely intersection in the Archaea. In the case of the Bacteria, reduction in the amount of ‘missing data’ also produced little effect on the distribution of log 10 Bayes factors (*r*^2^ = 0.953, *p* < 0.0001), however the position of the most likely intersection moved from the base of the mesophilic methanogens and Halobacteria to the node which separates the mesophilic methanogens, Halobacteria and Archaeoglobales from the rest of the tree. This position is still deeply embedded within the methanogenic Archaea.

## 4. Discussion

The study of the origin and evolution of life on the Earth is fast moving and iterated by the constant and exponential increase in available data from genome sources. Hence, it is not surprising that few data unify the multitude of hypotheses, which exist in the literature. However, some crucial details impose directionality on the probable course of events. The most important of these facts is that the common ancestor of all sampled extant eukaryotes contained an endosymbiont which originated from an α-proteobacterium [4,6–8]. This undisputed fact establishes a temporal order which requires that the major lineages of the Bacteria arose before the appearance of the last common ancestor of all extant eukaryotes. While the identity of the endosymbiont is not debated, the identity of the host is still in contention. Two main themes pervade the majority of hypotheses which explain the origin of the host cell: one stipulates that the host cell was a member of the Archaea (e.g. [7,12,17,18]). The other stipulates that the host cell was a pre-eukaryote (and possibly even pre-Archaea) ancestor [32,33]. Hypotheses which adopt this second view propose that the Archaea and modern day eukaryotes are sister groups, whose evolutionary histories are entwined for a time following the split from Bacteria. The variant forms of both themes rely on either phylogenetic reconstructions or biochemical/gene-presence synapomorphies or a combination of both.

We undertook to contribute to the understanding of these fundamental early evolutionary events using an alternative and novel approach. Using sensitive homologue-finding algorithms and highly conservative criteria for data selection, we identified 3537 discrete orthologue groups distributed throughout the Archaea. Interestingly, this detection/non-detection data are informative enough to recapture the majority of the phylogenetic relationships of the Archaea previously captured by multi-gene protein sequence phylogenetic inferences and rRNA analysis. By using the protein sequence contained within the 3537 DOGs, we are able to produce a phylogeny which is robust under Bayesian, maximum likelihood and quartet analysis methods. This phylogeny is also supported—in multiple independent re-sampled tree inferences—by three different and widely used models of amino acid substitution. Using this information-rich dataset, we independently interrogated the intersection of our robust archaeal phylogeny with both the eukaryotes and the Bacteria using Bayesian and maximum-likelihood methods. We show that this novel approach overcomes issues which arise from assuming a particular model of sequence evolution and hence, as an alternative to previous studies which have focused on finding the best models to fit the data, we demonstrate that our analyses are both method and model independent.

Our data show that the most likely intersection of the archaeal and bacterial trees resides within the archaeal methanogens rather than between the Crenarchaea and Euryarchaea as previously proposed [13,14]. We provide support for this by an additional analysis in which all DOGs found only in the methanogenic Archaea and any Bacteria have been removed, thereby reducing the phylogenetic signal attributable to lateral gene transfer signal. We also provide support by comparative analysis of the eukaryotic DOGs which are found only in Archaea and hence must have archaeal origins with the eukaryotic DOGs, which can be found in both Archaea and Bacteria and hence may have bacterial or archaeal origins. Our findings provide strong molecular support for the hypothesis that methanogenesis was the ancestral form of energy metabolism in the very first free-living Archaea [34,35]. These same theories propose that acetogenesis was the ancestral form of energy metabolism in the first Bacteria [34,35] and both theories find strong support for the ancient origins of these biochemistries from geological evidence isolated from 3.45 billion year old hydrothermal precipitates [63].

As all eukaryotes are a derived domain which arose later in the evolution of cellular life, the presence of a distinct intersection with the Archaea necessitates that this intersection occurred later than the intersection between the Archaea and the Bacteria. This implies that the major lineages of Archaea had also already diversified before the emergence of the last common ancestor of all extant eukaryotes. This observation is probably incompatible with hypotheses which propose that the Archaea and Eukaryota are sister groups, but rather stipulates that the eukaryotes themselves are derived from a particular branch of the Archaea. Moreover, this analysis specifies that the host cell which adopted the α-proteobacterium endosymbiont, and is hence the ancestor of all extant eukaryotes, diverged from the Archaeal line somewhere around the split of the Thaumarchaea from the Euryarchaea. Indeed from the genome data currently available, and in line with previous reports [56], it appears that the most likely eukaryote ancestor was either a member of or a sister group to the Thaumarchaea.
